# Supplementary material for: Biology of Perseverative Negative Thinking: The Role of Timing and Folate Intake
Source: Nutrients. 2021 Dec 8;13(12):4396. doi: 10.3390/nu13124396 (PMC8703428; doi:10.3390/nu13124396)
Supplement: Supplementary file 1 [file nutrients-13-04396-s001.zip › SupplementaryFileS1.pdf]

Biology of perseverative negative thinking: the role of timing and folate intake

Nora Eszlari 1,2,*, Bence Bruncsics 3, Andras Millinghoffer 2,3, Gabor Hullam 3,4, Peter Petschner 1,4,5, Xenia Gonda 2,4,6, Gerome Breen 7,8, Peter Antal 3, Gyorgy Bagdy 1,2,4, John Francis William Deakin 9 and Gabriella Juhasz 1,4,10

1 Department of Pharmacodynamics, Faculty of Pharmacy, Semmelweis University, Nagyvárad tér 4, H-1089 Budapest, Hungary; petschner.peter@pharma.semmelweis-univ.hu (P.P.); bagdy.gyorgy@pharma.semmelweis-univ.hu (G.B.); juhasz.gabriella@pharma.semmelweis-univ.hu (G.J.)

2 NAP-2-SE New Antidepressant Target Research Group, Hungarian Brain Research Program, Semmelweis University, Nagyvárad tér 4, H-1089 Budapest, Hungary; milli@mit.bme.hu (A.M.); gonda.xenia@med.semmelweis-univ.hu (X.G.)

3 Department of Measurement and Information Systems, Budapest University of Technology and Economics, Magyar tudósok krt. 2, H-1521 Budapest, Hungary; bruncsics@mit.bme.hu (B.B.); gabor.hullam@mit.bme.hu (G.H.); antal@mit.bme.hu (P.A.)

4 MTA-SE Neuropsychopharmacology and Neurochemistry Research Group, Hungarian Academy of Sciences, Semmelweis University, Nagyvárad tér 4, H-1089 Budapest, Hungary

5 Bioinformatics Center, Institute for Chemical Research, Kyoto University, Gokasho, Uji, Kyoto 611-0011, Japan

6 Department of Psychiatry and Psychotherapy, Semmelweis University, Gyulai Pál utca 2, H-1085 Budapest, Hungary

7 Social, Genetic and Developmental Psychiatry Centre, Institute of Psychiatry, Psychology and Neuroscience, King's College London, Memory Lane, London SE5 8AF, UK; gerome.breen@kcl.ac.uk

8 UK National Institute for Health Research (NIHR) Maudsley Biomedical Research Centre (BRC), , London SE5 8AF, UK

9 Division of Neuroscience and Experimental Psychology, Faculty of Biology, Medicine and Health, The University of Manchester, M13 9PL Oxford Road, Manchester, UK; Bill.Deakin@manchester.ac.uk

10 SE-NAP 2 Genetic Brain Imaging Migraine Research Group, Hungarian Brain Research Program, Semmel-weis University, Nagyvárad tér 4, H-1089 Budapest, Hungary

* Correspondence: eszlari.nora@pharma.semmelweis-univ.hu; Tel.: +36-1459-1500 (ext. 56153)

Supplementary File 1

Methods

Phenotypes

In UK Biobank, current depression score was determined by the sum of answer scores on the following questions: "Over the past two weeks, how often have you felt down, depressed or hopeless?" (data field “2050”), "Over the past two weeks, how often have you had little interest or pleasure in doing things?" (data field “2060”), "Over the past two weeks, how often have you felt tense, fidgety or restless?" (data field “2070”), and "Over the past two weeks, how often have you felt tired or had little energy?" (data field “2080”).

In UK Biobank, a cut-off point of a daily 200 µg for a healthy level of folate intake was based on recommendation of the British government, for the general population over 19 years old (https://assets.publishing.service.gov.uk/government/uploads/system/uploads/attachment_data/file/618167/government_dietary_recommendations.pdf), ensuring that the needs of 97.5% of this population are met (https://www.nutrition.org.uk/attachments/article/907/Nutrition%20Requirements_Revised%20August%202019.pdf).

Genotyping, imputation and genomic quality control

Info and certainty thresholds for imputed SNPs in UK Biobank were both set to 0.9 [1]. Imputation procedure within NewMood has been detailed elsewhere [2].

In both UK Biobank and NewMood, the same QC steps and thresholds were applied regarding minor allele frequency (MAF) (0.01), SNP and subject missingness (iteratively 0.1, 0.05, and 0.01), Hardy-Weinberg equilibrium test ($p \geq 1x10^{-5}$), and linkage disequilibrium (LD) pruning (R^2 of 0.2) before further steps of kinship check, sex check, heterozygosity outlier detection, and before principal component analysis. All of these steps have been detailed elsewhere [2]. To provide a strict selection of unrelated participants, a filter of “--ibd 0.1875” was applied in NewMood [2], and “--kin 0.044” in UK Biobank [3].

In both databases, analyses were restricted to autosomal chromosomes.

Analyses

In logistic regression models run in Plink v1.9, additive effect of each of 6 013 935 SNPs was tested in the whole study sample, 5 992 065 SNPs were analyzed in the suboptimal folate intake group, and 6 012 113 SNPs in the optimal folate intake group. These numbers entail a $p \leq 8.30x10^{-9}$ Bonferroni-corrected significance threshold within all three groups, and thus a $p \leq 1.38x10^{-9}$ threshold if considering all of the six analyses. Sex, age, top ten principal components of the genome, current depression level, lifetime depression status and genotype array (as a binary variable) were included as predictors in all models. A MAF filter of 0.01 was also applied in every run.

Within FUMA, MAGMA v1.07 [4] was used for gene-based and gene set-based analyses, and for gene property analysis that assesses tissue specificity.

MAGMA gene-based test was the aggregation of SNP-based p-values to gene-based p-values through a SNP-wise mean model. This test assigned SNPs to protein-coding genes based on position, with gene boundaries extended by 10 000 base pairs. We considered $p \leq 4.36x10^{-7}$ as significant for genes: the nominal p-level 0.05 divided by 114 642, which equals to 19 107 genes by six analyses.

MAGMA gene set test meant regressing transformed gene p-value on the gene set indicator variable (whether or not the gene is member of that set), covarying also minor allele count, gene size, LD between SNPs and between close genes. 15 496 gene sets from MsigDB v7.0 were tested: 5 500 C2 curated gene sets and 9 996 C5 GO terms. In our analyses, this entailed 15 485 gene sets and thus a Bonferroni-corrected p-value threshold of $3.23x10^{-6}$, and a $5.38x10^{-7}$ for all of the six tests.

In MAGMA, to explore tissue specificity of upregulated genes that are relevant in either “rumination” or “worry”, converted gene p-value was regressed on gene expression of a given tissue, covarying technical confounders, and average expression across all tissues in the data set. Expression databases for testing were the 30 general tissue types of GTEx v8 [5], and the 11 general developmental stages of BrainSpan’s developmental brain samples [6]. These 41 tissues by the six tests entailed a $2.03x10^{-4}$ Bonferroni-corrected p-value threshold.

To overcome limitations of only position-based assignment and only to protein-coding genes within MAGMA, additional gene mapping was performed with “SNP2GENE” function of FUMA.

Genomic risk loci for each of the six analyses were defined so that each one should have a lead SNP of $p \leq 1x10^{-5}$, and should have SNPs of $p \leq 0.05$ and an $R^2 \geq 0.5$ with the lead SNP. SNPs of these genomic risk loci were then mapped to genes of Ensembl v92 via three different ways. 1.) A SNP was mapped to a gene if residing within gene boundaries extended by 10 000 base pairs. 2.) It was mapped to a gene if it has been associated with that gene’s expression at a false discovery rate (FDR) $p \leq 0.05$ in any tissue and any expression quantitative trait loci (eQTL) database included in FUMA. 3.) It was mapped to a gene if it significantly (FDR $p \leq 1x10^{-6}$) takes part in a 3D chromatin interaction with promoter region of the gene in any database.

These mapped genes were then further analyzed with hypergeometric tests if overrepresented in a predefined gene set. In these enrichment analyses implemented in “GENE2FUNC” function of FUMA, adjusted p-value cut-off was an FDR $p<0.05$ within each subcategory of gene sets (such as within GO biological processes). In this paper only the two subcategories (chemical and genetic perturbations, CGP; and canonical pathways, CP) of MsigDB C2, and the three subcategories (biological process, BP; cellular component, CC; and molecular function, MF) of MsigDB C5 gene set collections were considered, to provide a comparability with MAGMA gene set results. The weakness of this enrichment test is that it does not correct for LD between close genes within the gene set.

To test UK Biobank polygenic risk scores (PRSs) in NewMood, analyses were restricted to SNPs present in both UK Biobank and NewMood, and matching in both minor and major alleles between them. Clumping was performed with a 250 kilobase wide window to both sides of, and an $R^2 \geq 0.2$ with, the index SNP. From each of UK Biobank’s six SNP-level logistic regression analyses, top risk SNPs were included according to numerous p-value thresholds. Then, for every NewMood participant, each risk allele’s count multiplied by its effect size in UK Biobank was summed, and averaged by number of included alleles. Explanatory value of this PRS was then tested in an additive genetic model for standardized residuals of neuroticism, rumination, brooding and reflection scores. For each of the 24 analyses, we were interested in explanatory value of PRS in case of the best fit model, among many models with different p-value inclusion thresholds from UK Biobank GWAS results. To control for false positive results because of multiple testing, 10 000 permutations were performed for each of these 24 best-fit models, and models with the resulted empirical $p \leq 0.05$ were considered significant.

PRS calculation with PRSice-2 was also applied to test the explanatory value of top SNPs within suboptimal folate intake group for the same perseverative negative thinking item within optimal folate intake group, and vice versa. Regarding clumping, polygenic score calculation and testing for standardized residuals, as well as permutation, the same procedure was applied as in PRS analyses for NewMood phenotypes.

In all regression models calculating standardized residuals for further PRS testing, sex, age, top ten principal components of the genome, current depression level, and lifetime depression status were included as predictors. In UK Biobank analyses, genotype array, as a binary variable, was an additional covariate. For

each subscale of rumination in NewMood, the other subscale was also a covariate. A MAF filter of 0.01 was also applied in every PRS testing.

Results

Descriptive statistics

Frequencies or means for the variables used in UK Biobank are displayed in ***Supplementary Table 1***. ***Supplementary Table 2*** (direction of effects not shown) depicts relationships between outcome and predictor variables of further logistic regression models that will test an additive genetic effect. Folate intake was inversely related to “worry” item, but unrelated to “rumination” item. Both items were in a positive association with both depression phenotypes, and were negatively associated with age in all three groups. Also in all three groups, there were more “ruminators” and “worriers” among females than among males.

		Whole sample		Suboptimal folate intake group		Optimal folate intake group	
		**Count**	**%**	**Count**	**%**	**Count**	**%**
**"Do you worry too long after an embarrassing experience?"**	**No**	37228	51.3	5458	51.3	31770	51.3
	**Yes**	35393	48.7	5180	48.7	30213	48.7
**"Are you a worrier?"**	**No**	32831	45.2	4711	44.3	28120	45.4
	**Yes**	39790	54.8	5927	55.7	33863	54.6
**Sex**	**Male**	33778	46.5	4174	39.2	29604	47.8
	**Female**	38843	53.5	6464	60.8	32379	52.2
**Lifetime depression status**	**Never depressed**	68772	94.7	9968	93.7	58804	94.9
	**Ever depressed**	3849	5.3	670	6.3	3179	5.1
**Folate intake**	**Below 200 µg/day**	10638	14.6				
	**Above 200 µg/day**	61983	85.4				
		**Mean**	**S.E.M.**	**Mean**	**S.E.M.**	**Mean**	**S.E.M.**
**Age**		56.762	0.0293	55.544	0.0779	56.971	0.0315
**Current depression score (scoring 1-4)**		1.368	0.0018	1.430	0.0055	1.358	0.0019

***Supplementary Table S1.* Descriptive statistics for the whole sample and separate subgroups of the investigated UK Biobank sample (Application number 1602). S.E.M.: standard error of mean.**

			Folate intake	Sex	Lifetime depression status		Current depression score	Age
**Whole sample**	**"Do you worry too long after an embarrassing experience?"**	**χ2**	0.009	1303.060	532.132	**t**	-64.160	10.116
		**df**	1	1	1	**df**	63773.048	72517.065
		**p**	0.923	2.44E-285	9.71E-118	**p**	0.000E+00	4.873E-24
	**"Are you a worrier?"**	**χ2**	4.296	1933.272	901.283	**t**	-80.901	4.226
		**df**	1	1	1	**df**	68102.199	69659.396
		**p**	0.038	0.000E+00	5.162E-198	**p**	0.000E+00	2.377E-05
**Suboptimal folate intake group**	**"Do you worry too long after an embarrassing experience?"**	**χ2**		248.064	135.450	**t**	-25.973	3.814
		**df**		1	1	**df**	9223.191	10631.170
		**p**		6.863E-56	2.633E-31	**p**	1.348E-143	1.374E-04
	**"Are you a worrier?"**	**χ2**		288.041	185.930	**t**	-31.150	3.323
		**df**		1	1	**df**	10100.921	10636.000
		**p**		1.329E-64	2.459E-42	**p**	1.760E-203	0.001
**Optimal folate intake group**	**"Do you worry too long after an embarrassing experience?"**	**χ2**		1065.694	402.078	**t**	-58.845	9.397
		**df**		1	1	**df**	54637.939	61981.000
		**p**		9.443E-234	1.944E-89	**p**	0.000E+00	5.784E-21
	**"Are you a worrier?"**	**χ2**		1641.653	715.291	**t**	-74.852	3.051
		**df**		1	1	**df**	58062.392	59562.400
		**p**		0.000E+00	1.415E-157	**p**	0.000E+00	0.002

Supplementary Table S2. **Relationships between the two perseverative negative thinking items and the other variables, within the whole sample and separate subgroups of the investigated UK Biobank sample (Application number 1602). All relationships are significant, except for that of "rumination" and folate intake. χ^2: Pearson chi-square, t: t-value, df: degrees of freedom, p: p-value.**

Frequencies or means for the variables used in NewMood are listed in ***Supplementary Table 3***. Note that the NewMood sample is younger, more depressed and more predominated by females than the UK Biobank sample. In ***Supplementary Table 4*** we can see relationships between outcome and predictor variables of further linear regression models that will yield standardized residuals in NewMood, for which we will then test explanatory value of polygenic risk scores (PRS) composed of UK Biobank's top risk variants. All four outcome scales had positive associations with both depression phenotypes, and females scored higher than males on all four of them. Age showed a negative correlation with the three rumination phenotypes, but was unrelated to neuroticism. Brooding and reflection had an r=0.487 Pearson correlation with each other ($p=1.252x10^{-104}$).

		**Count**	**%**
**Sex**	**Male**	501	28.7
	**Female**	1245	71.3
**Lifetime depression status**	**Never depressed**	1034	59.2
	**Ever depressed**	712	40.8
		**Mean**	**S.E.M.**
**Age**		32.526	0.2506
**Neuroticism score (scoring 1-5)**		3.133	0.0220
**Rumination score (scoring 1-4)**		2.118	0.0137
**Brooding score (scoring 1-4)**		2.146	0.0161
**Reflection score (scoring 1-4)**		2.091	0.0157
**BSI depression score (scoring 0-4)**		0.845	0.0220

Supplementary Table S3. **Descriptive statistics for NewMood. Range of possible values of scale scores are also indicated. S.E.M.: standard error of mean, BSI: Brief Symptom Inventory.**

		**Sex**	**Lifetime depression status**		**BSI depression score**	**Age**
**Neuroticism score**	**t**	-9.987	-24.800	**r**	0.621	-0.001
	**df**	1744	1744			
	**p**	7.071E-23	1.501E-116	**p**	1.872E-186	0.962
**Rumination score**	**t**	-8.977	-19.808	**r**	0.588	-0.072
	**df**	1744	1407.454			
	**p**	7.007E-19	3.171E-77	**p**	8.237E-163	0.003
**Brooding score**	**t**	-8.458	-19.977	**r**	0.627	-0.059
	**df**	1010.888	1336.000			
	**p**	9.420E-17	6.747E-78	**p**	1.465E-191	0.014
**Reflection score**	**t**	-7.266	-13.466	**r**	0.384	-0.065
	**df**	1744	1744			
	**p**	5.556E-13	2.156E-39	**p**	2.564E-62	0.007

Supplementary Table S4. **Relationships between scale scores and other variables in NewMood. t: t-value, df: degrees of freedom, r: Pearson correlation coefficient, p: p-value.**

SNP-based results

Supplementary Figure S1 displays significance of SNP-level associations separately within the six analyses.

a) Rumination, whole sample

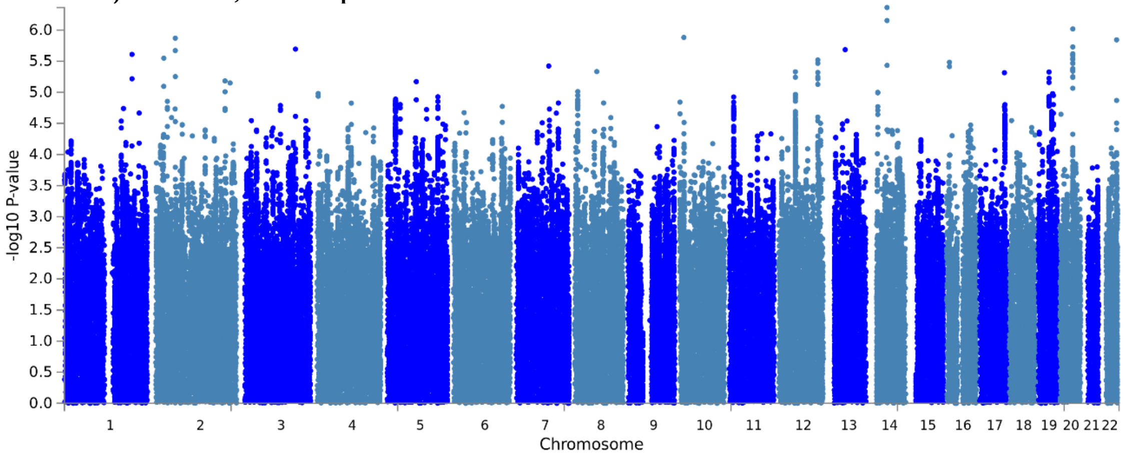

b) Worry, whole sample

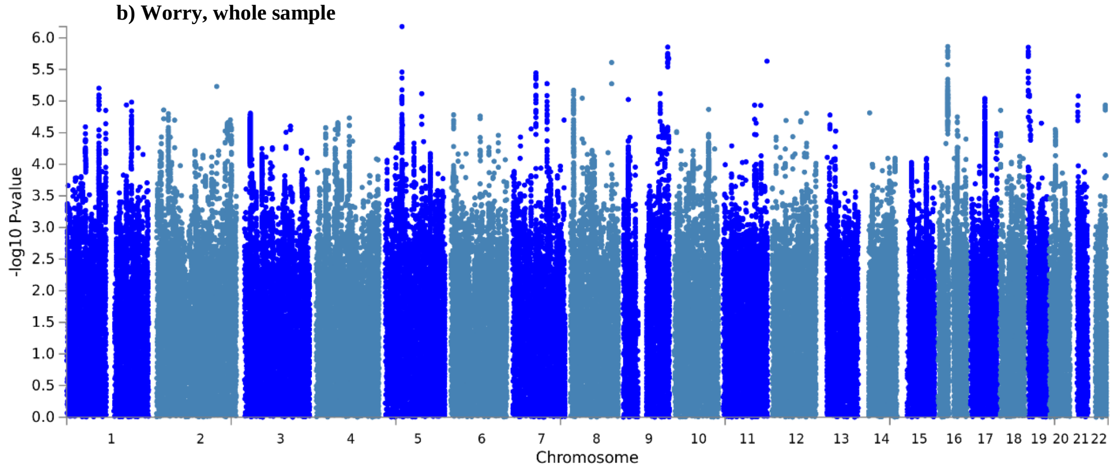

c) Rumination, suboptimal folate

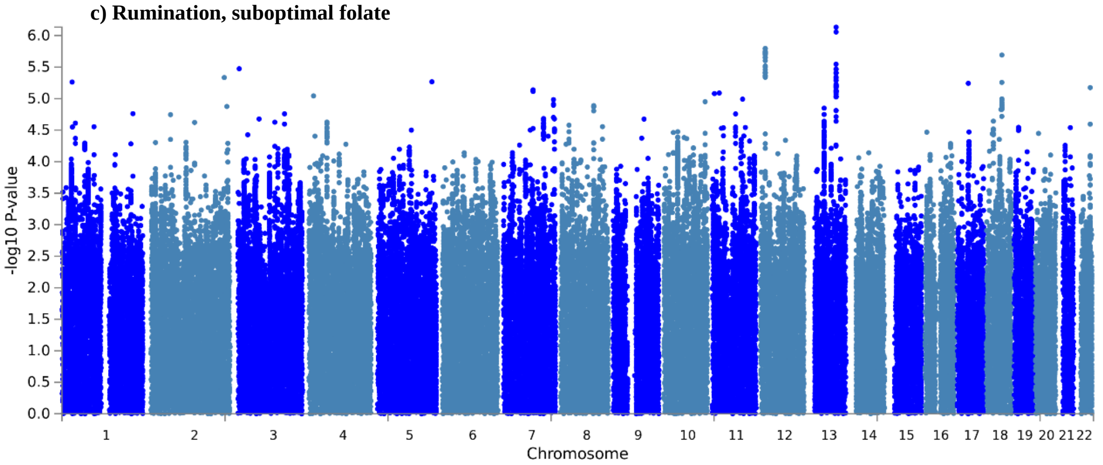

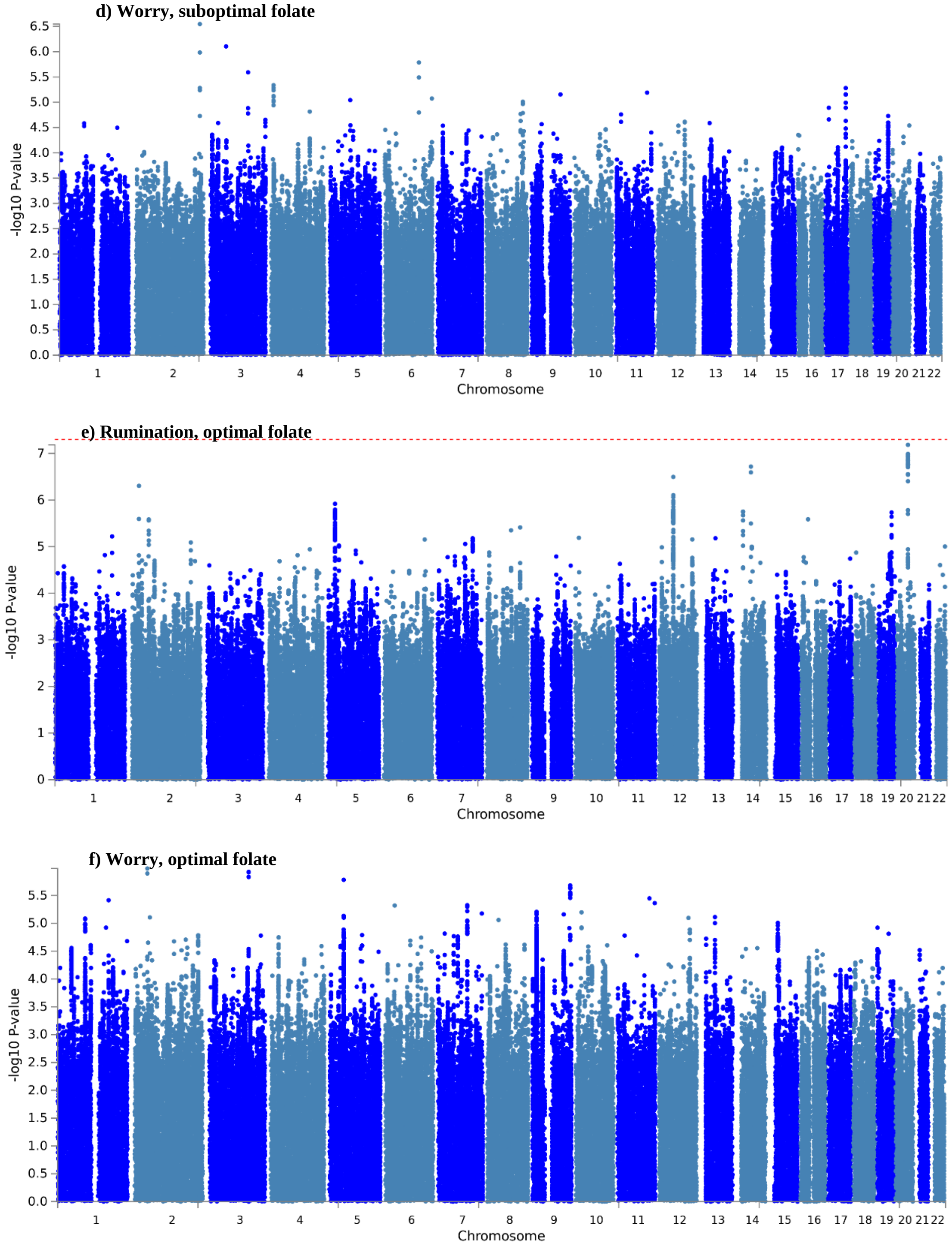

Supplementary Figure S1. **Manhattan plots of SNP-based tests for "rumination" and "worry" items in the whole study sample (1a and 1b), in the suboptimal folate intake group (1c and 1d), and in the optimal folate intake group (1e and 1f). -log10 of p-value is displayed in function of genomic position. Red line denotes significance threshold corrected only within one analysis. SNP: single-nucleotide polymorphism.**

QQ plots for each of the six SNP-based analyses, with genomic inflation factor lambda (λ), are aligned in ***Supplementary Figure S2***.

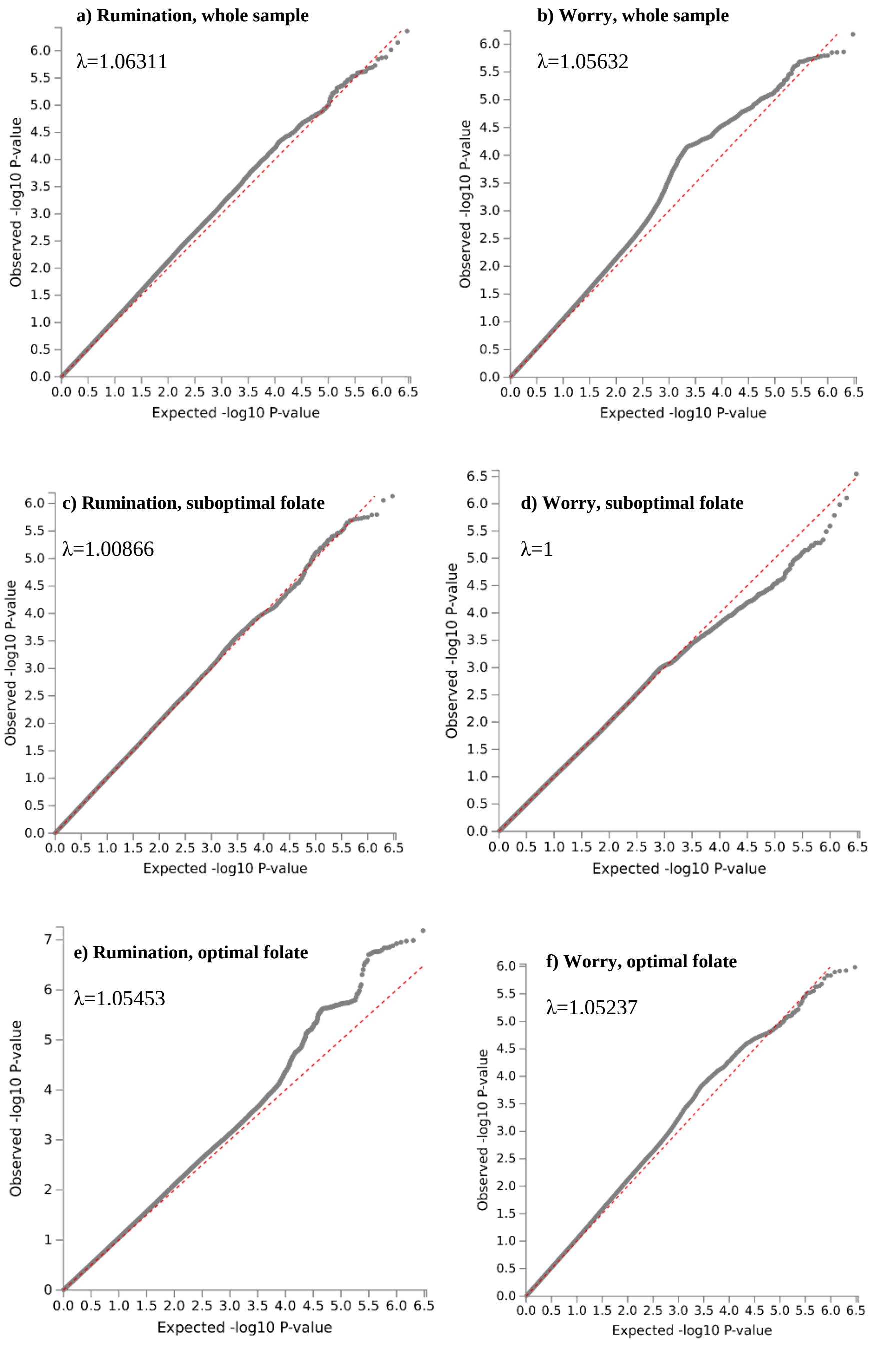

Supplementary Figure S2. **QQ plots of SNP-based tests for "rumination" and "worry" items in the whole study sample (2a and 2b), in the suboptimal folate intake group (2c and 2d), and in the optimal folate intake group (2e and 2f). Observed -log10 p-values are displayed in function of their expected values. SNP: single-nucleotide polymorphism.**

MAGMA’s gene-based results

Manhattan plots of MAGMA’s gene-based tests are displayed in ***Supplementary Figure S3***.

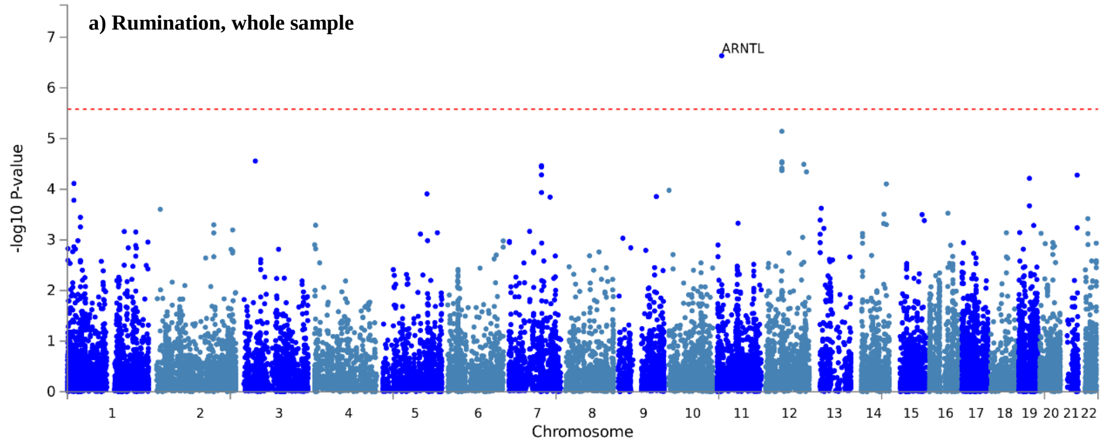

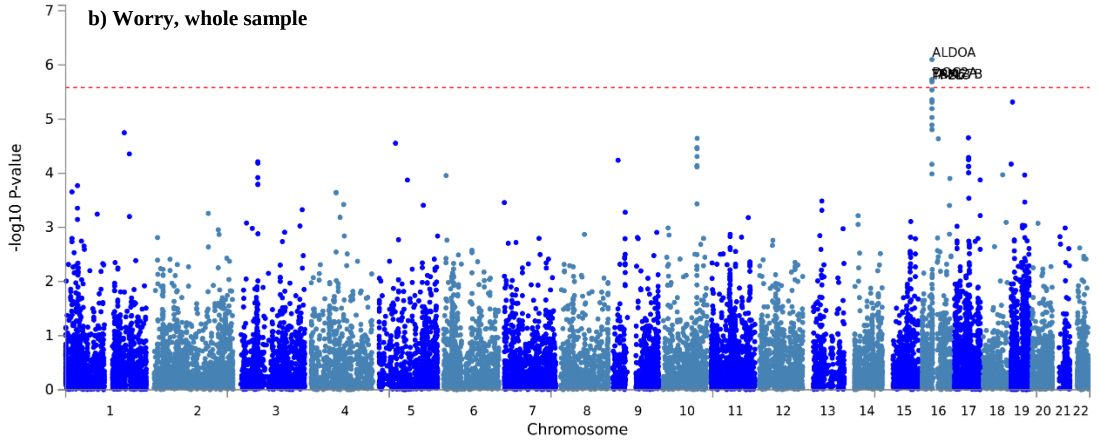

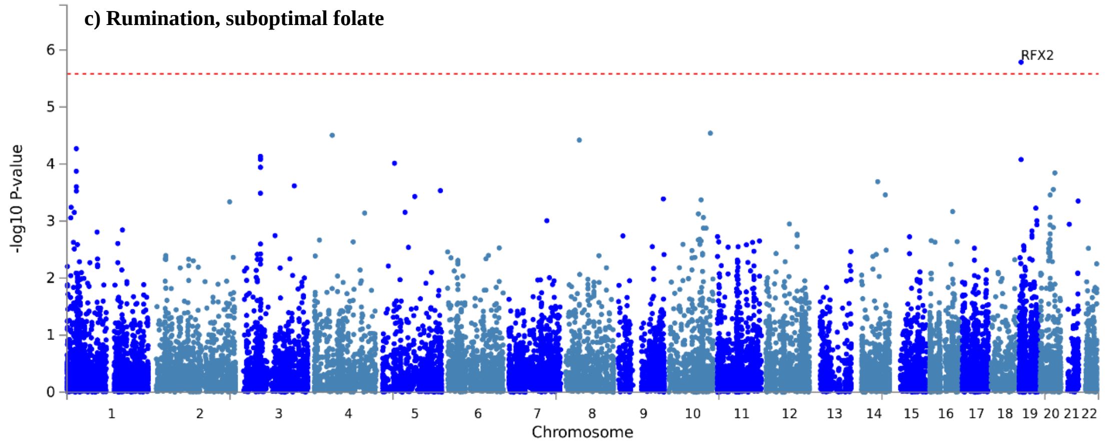

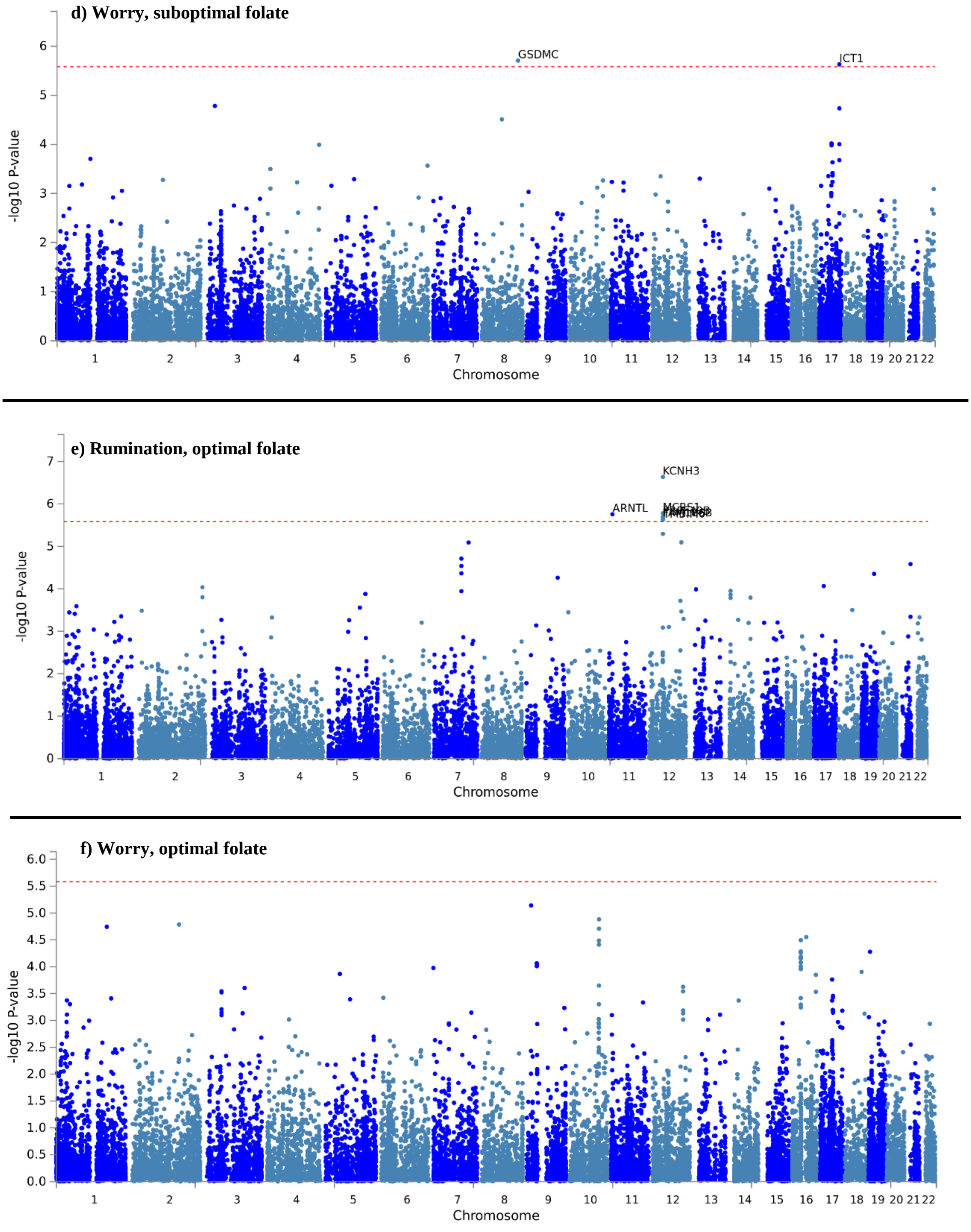

Supplementary Figure S3. **Manhattan plots of gene-based tests for "rumination" and "worry" items in the whole study sample (3a and 3b), in the suboptimal folate intake group (3c and 3d), and in the optimal folate intake group (3e and 3f). -log10 of p-value is displayed in function of genomic position. Red line denotes significance threshold corrected only within one analysis.**

Supplementary Figure S4 displays QQ plots for the six gene-based analyses, performed with MAGMA v1.07 within FUMA.

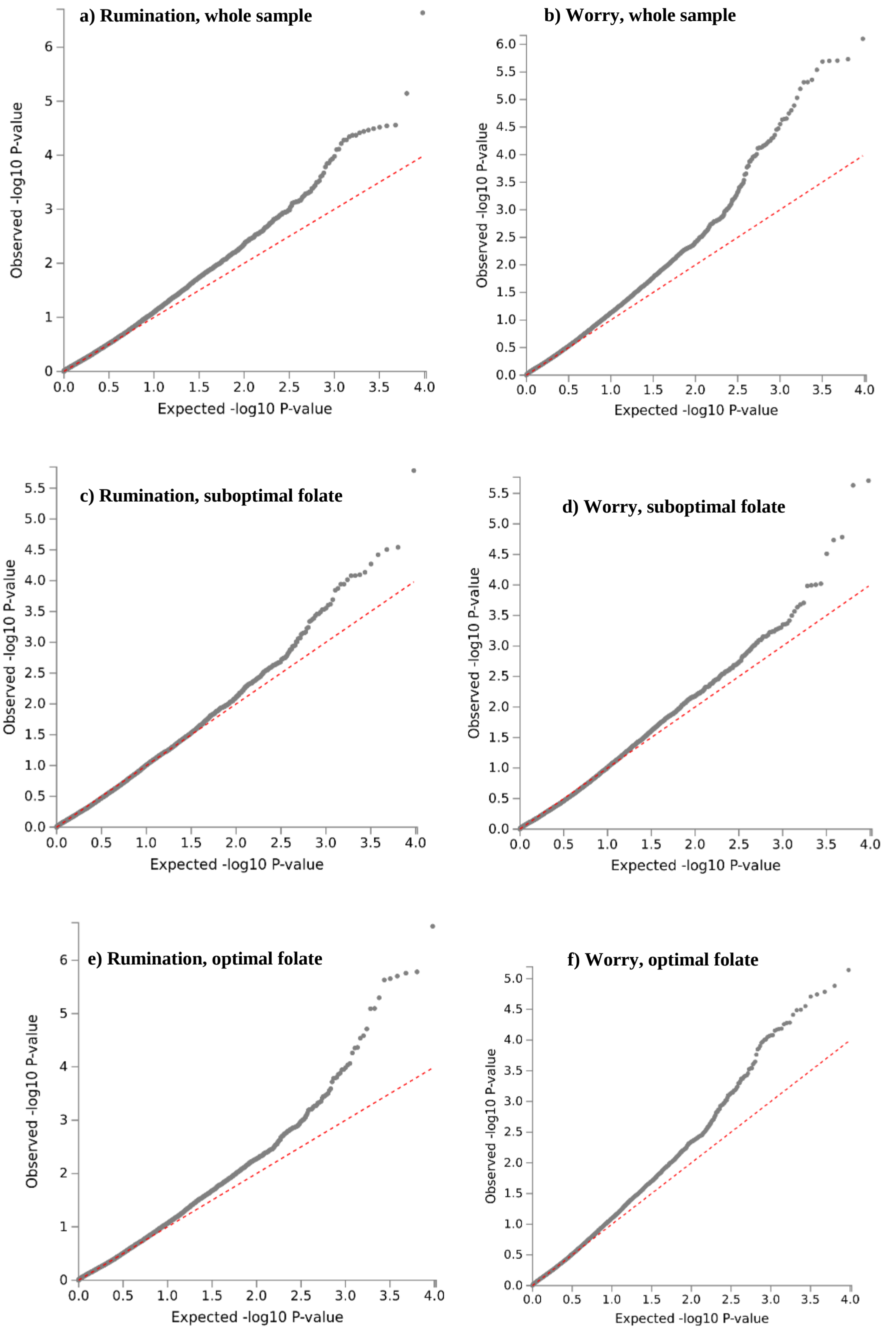

Supplementary Figure S4. **QQ plots of gene-based tests for "rumination" and "worry" items in the whole study sample (4a and 4b), in the suboptimal folate intake group (4c and 4d), and in the optimal folate intake group (4e and 4f). Observed -log10 p-values are displayed in function of their expected values.**

<u>MAGMA's tissue-specific upregulation of genes relevant in "rumination" or "worry"</u>

Supplementary Figures S5 and S6 show results of gene property analyses, implemented with MAGMA analyses of FUMA, and exploring tissue-specific upregulation of genes that are relevant at gene-level in either "rumination" or "worry" in a given group.

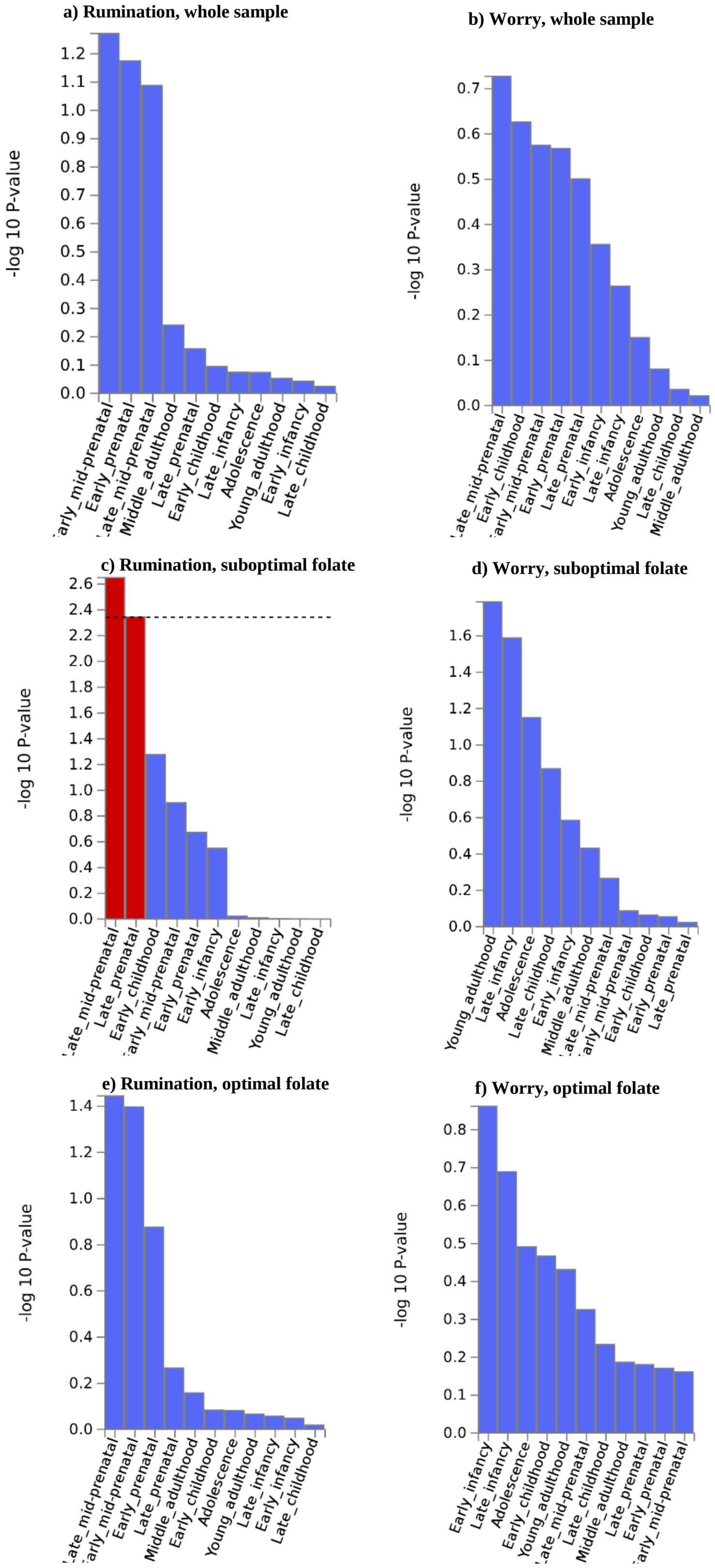

***Supplementary Figure S5.* Association of brain developmental stage-specific gene expression level with gene-based effect for "rumination" and "worry" items in the whole study sample (5a and 5b), in the suboptimal folate intake group (5c and 5d), and in the optimal folate intake group (5e and 5f). -log10 p-value is displayed for each of the 11 general developmental stages of BrainSpan's brain development samples.**

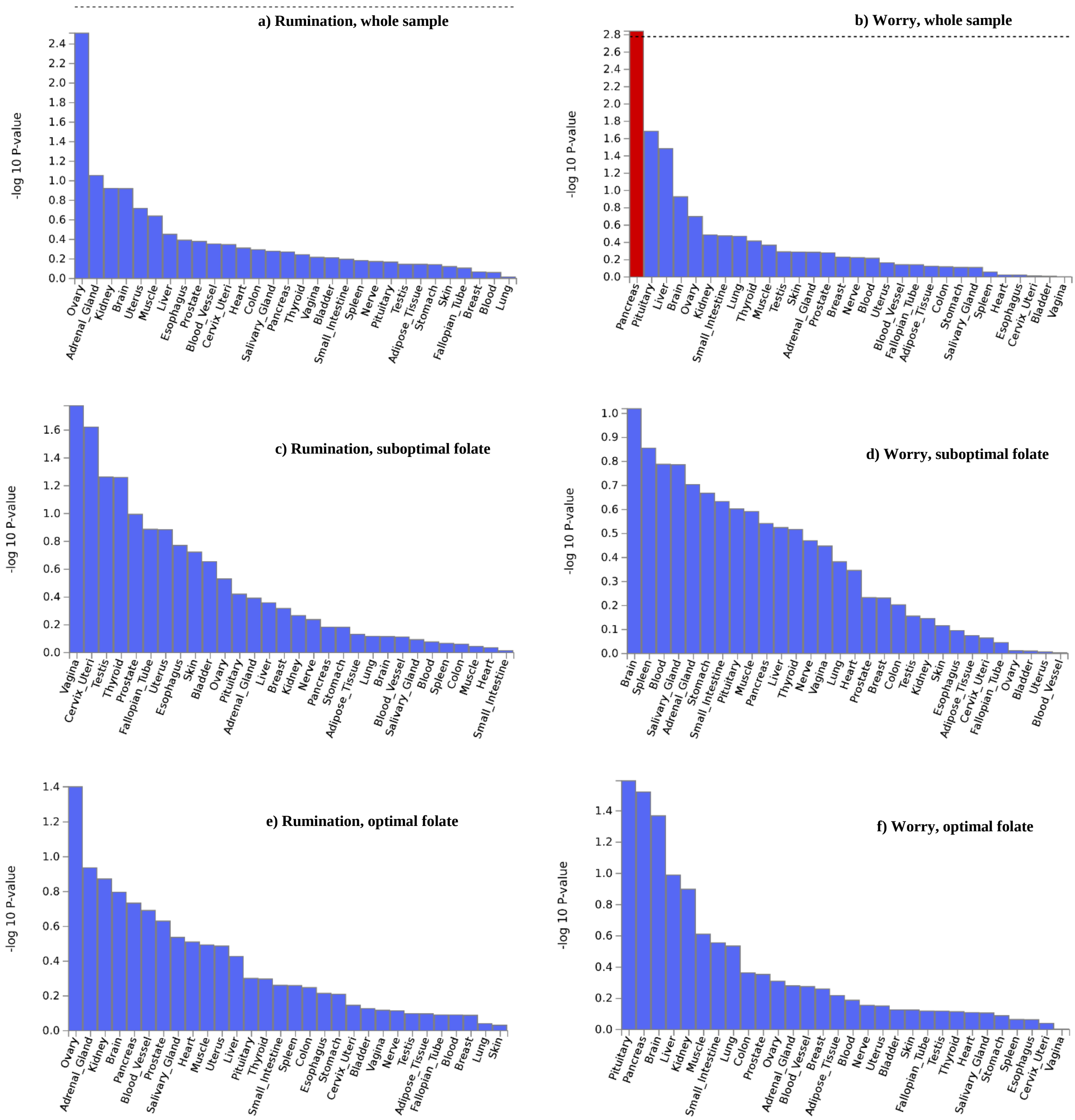

Supplementary Figure S6. **Association of tissue-specific gene expression level with gene-based effect for "rumination" and "worry" items in the whole study sample (6a and 6b), in the suboptimal folate intake group (6c and 6d), and in the optimal folate intake group (6e and 6f). -log10 p-value is displayed for each of the 30 general tissue types of GTEx v8.**

Genes mapped both by position and functional annotations, and their enrichment in MsigDB C2 and C5 gene sets

Genes mapped to the most significant SNPs by position, expression quantitative trait loci (eQTL) or 3D chromatin interaction were then investigated for enrichment in each gene set of multiple collections. ***Supplementary Figures S7-S12*** depict significantly enriched gene sets for each of the six analyses (two outcomes by three folate intake groups). The two subcategories (chemical and genetic perturbations: CGP, and canonical pathways: CP) of MsigDB C2 curated gene sets, and the three subcategories (biological process: BP, cellular component: CC, and molecular function: MF) of MsigDB C5 GO gene sets were considered, and a false discovery rate (FDR) p<0.05 significance criterion was applied within each of these five subcategories.

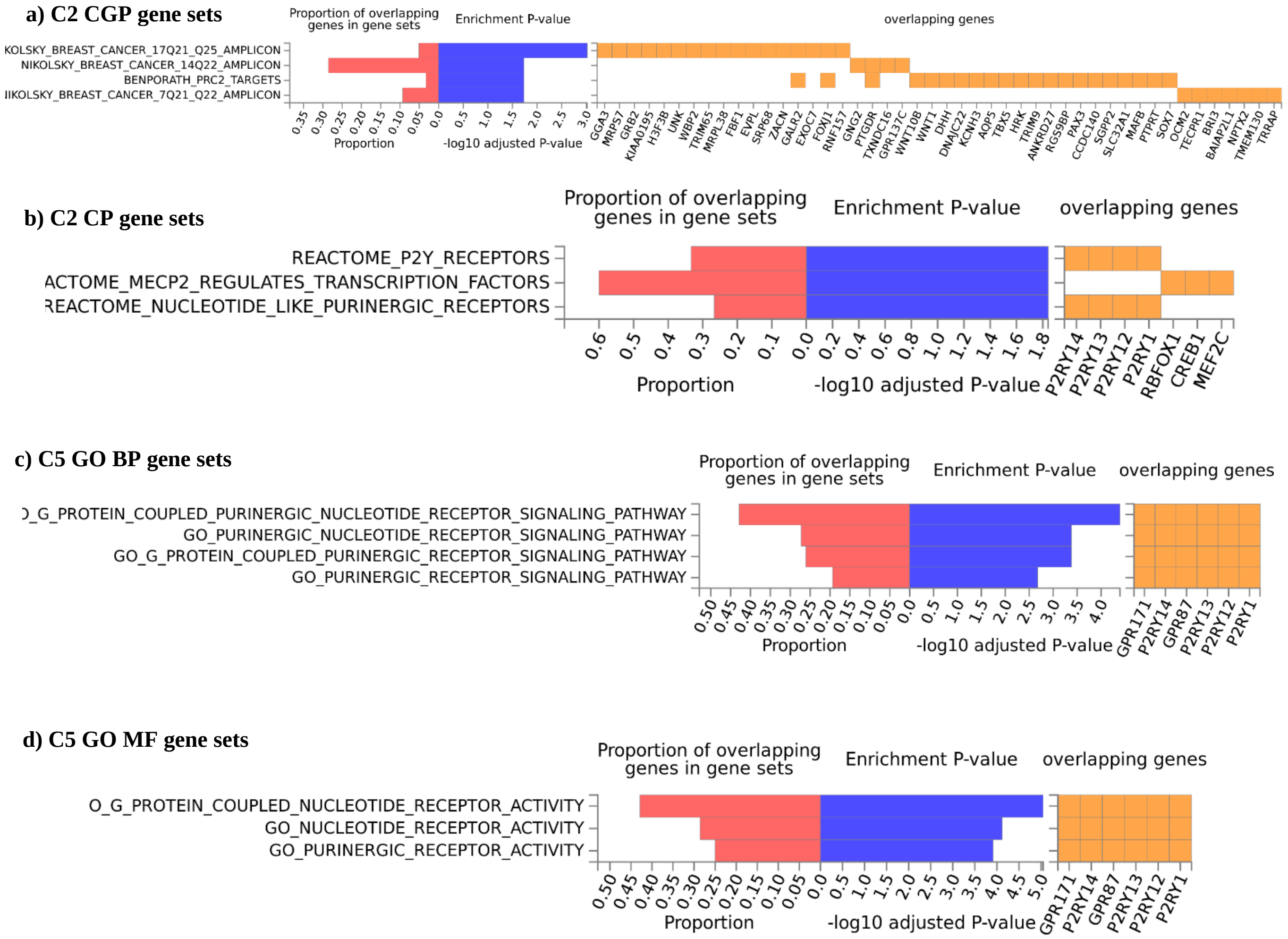

Supplementary Figure S7. **Significantly enriched gene sets for the "rumination" item in the whole study sample. CGP: chemical and genetic perturbations, CP: canonical pathways, BP: biological processes, MF: molecular function.**

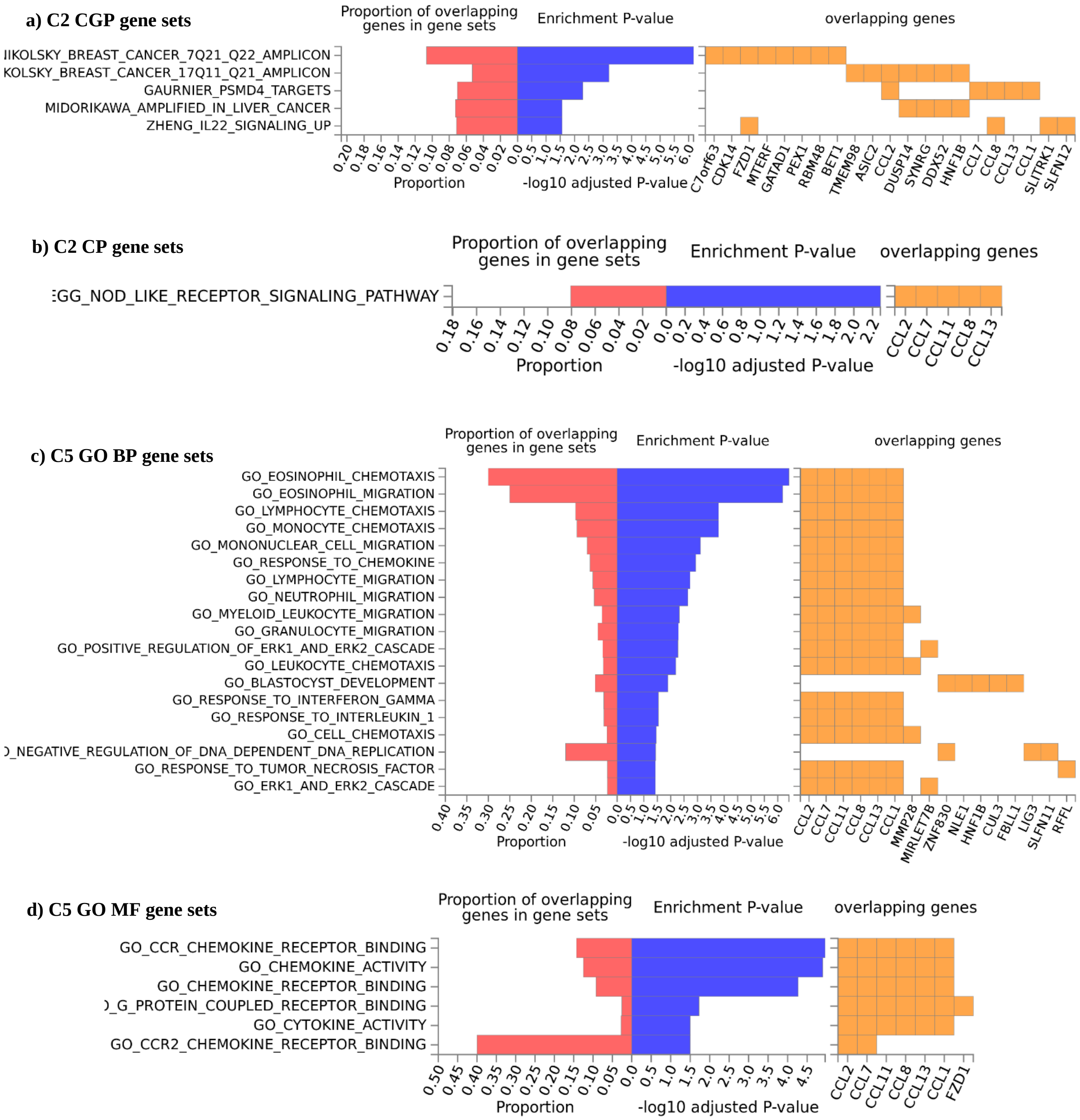

Supplementary Figure S8. **Significantly enriched gene sets for the "rumination" item in the suboptimal folate intake group. CGP: chemical and genetic perturbations, CP: canonical pathways, BP: biological processes, MF: molecular function.**

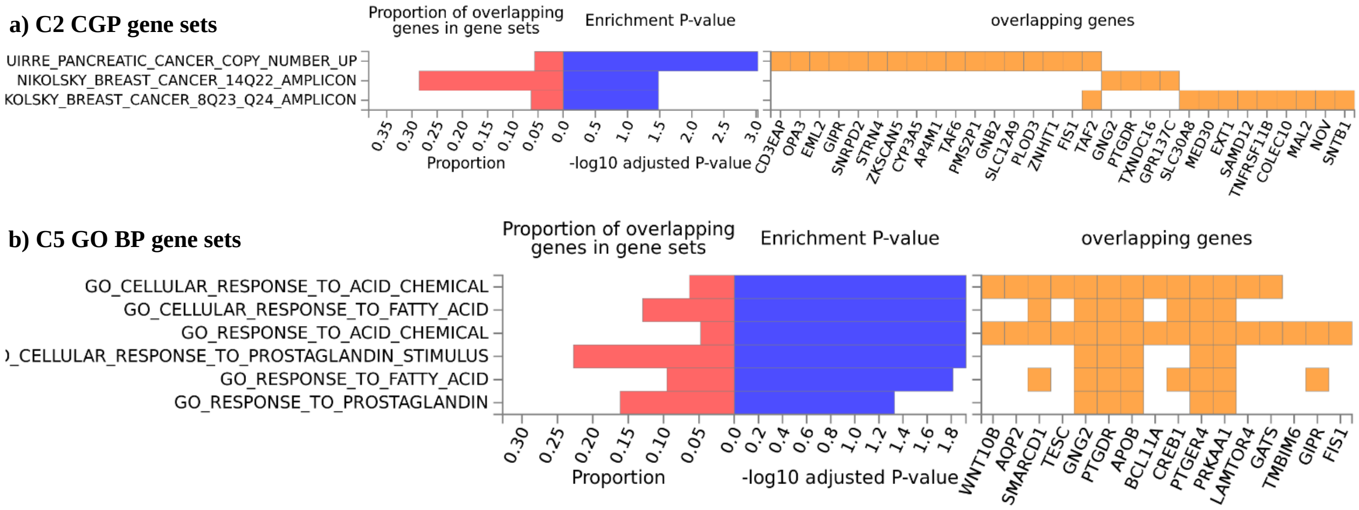

Supplementary Figure S9. **Significantly enriched gene sets for the "rumination" item in the optimal folate intake group. CGP: chemical and genetic perturbations, BP: biological processes.**

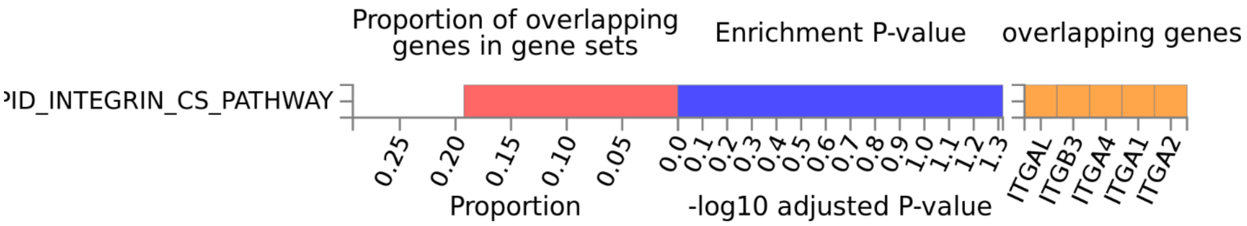

Supplementary Figure S10. **Significantly enriched gene set for the "worry" item in the whole study sample. It emerged in the "canonical pathways" subcategory of the C2 curated gene set collection.**

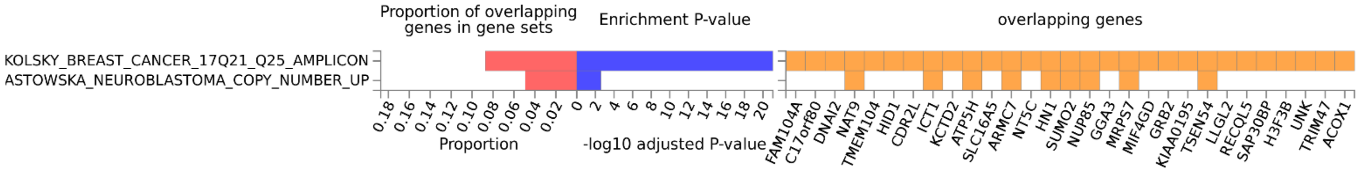

Supplementary Figure S11. **Significantly enriched gene sets for the "worry" item in the suboptimal folate intake group. They emerged in the "chemical and genetic perturbations" subcategory of the C2 curated gene set collection.**

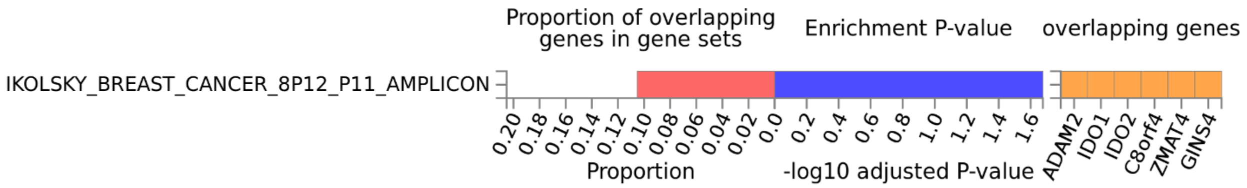

***Supplementary Figure S12.* Significantly enriched gene set for the “worry” item in the optimal folate intake group. It emerged in the “chemical and genetic perturbations” subcategory of the C2 curated gene set collection.**

Explanatory value of UK Biobank's risk SNPs in NewMood's phenotypes

From the 24 polygenic risk score (PRS) analyses that tested the explanatory role of UK Biobank's risk SNPs for NewMood's full-scale phenotypes, only one result survived permutation: the most significant risk SNPs for "rumination" item in the optimal folate group significantly explained 0.56% of variance in the rumination scale (***Supplementary Figure S13***).

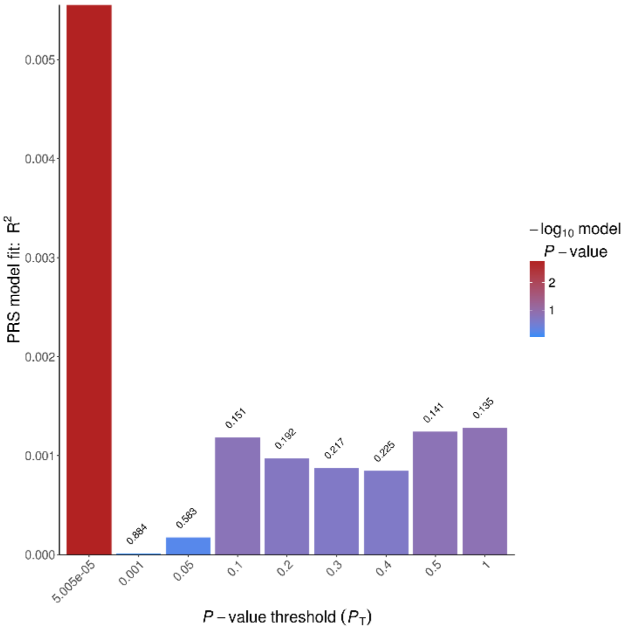

Supplementary Figure S13. **Explanatory value of "rumination" risk SNPs within UK Biobank's optimal folate intake group, for NewMood's rumination scale. Explained variance (R^2) in NewMood is displayed in function of p-value inclusion threshold in UK Biobank. Colors denote -log10 p-value of the model that regresses rumination scale on PRS, suggesting the highest explanatory value in case of the most significant few SNPs, and pointing to some shared genetics between "rumination" item and a widely used rumination scale across two different populations. PRS: polygenic risk score.**

Discussion

Comparison of present results to Nagel et al's finding

[Nagel, M., et al. (2018). "Item-level analyses reveal genetic heterogeneity in neuroticism." Nat Commun 9(1): 905.]

Nagel et al, 2018 [7] have conducted analyses on the same neuroticism items within the same UK Biobank database as our present work, and with a considerable overlap in analysis methods. However, since they were not interested in any aspect of diet, they were able to use a larger sample than ours: a size of around 365 000 - 370 000 participants. In contrast to our negative results, they could detect genome-wide significant SNPs for both items, which may be due to sample size, because their p-values increased with sample size. Consequently, regarding further levels, genes and gene sets, if only sample size did matter in our negative findings, our suggestive hits that do not survive correction would survive it in Nagel et al's. From our seven MAGMA gene hits for "rumination", only *ARNTL* survives multiple testing correction in Nagel et al's gene-based MAGMA results (Z-value=7.95), just as in our whole sample (Z-value=5.02). No MAGMA gene set for "rumination" survives correction in their results, similarly to ours. Just as in our results, none from our seven "worry" hit genes survives correction in their results, but, in contrast to ours, they do have other, surviving results. While we detected no significant MAGMA gene set for "worry", they revealed "axon part" gene set of C5 GO CC collection. These findings point to a more consistent genetic background of "rumination" than of "worry" across our study and Nagel et al's.

Our GWAS analyses for the two items differed from Nagel et al's also in that we covaried depression, but they covaried Townsend deprivation index [7]. Stability of "rumination's" genetic associations between the two studies, relative to "worry's", may suggest its strong endophenotypic relevance, independent of depressive symptoms, depression history, or sociodemographic factors.

References

1. Nagel, M.; Jansen, P.R.; Stringer, S.; Watanabe, K.; de Leeuw, C.A.; Bryois, J.; Savage, J.E.; Hammerschlag, A.R.; Skene, N.G.; Munoz-Manchado, A.B.; et al. Meta-analysis of genome-wide association studies for neuroticism in 449,484 individuals identifies novel genetic loci and pathways. *Nat Genet* **2018**, *50*, 920-927, doi:10.1038/s41588-018-0151-7.
2. Eszlari, N.; Millinghoffer, A.; Petschner, P.; Gonda, X.; Baksa, D.; Pulay, A.J.; Rethelyi, J.M.; Breen, G.; Deakin, J.F.W.; Antal, P.; et al. Genome-wide association analysis reveals KCTD12 and miR-383-binding genes in the background of rumination. *Transl Psychiatry* **2019**, *9*, 019-0454.
3. Bycroft, C.; Freeman, C.; Petkova, D.; Band, G.; Elliott, L.T.; Sharp, K.; Motyer, A.; Vukcevic, D.; Delaneau, O.; O'Connell, J.; et al. The UK Biobank resource with deep phenotyping and genomic data. *Nature* **2018**, *562*, 203-209.
4. de Leeuw, C.A.; Mooij, J.M.; Heskes, T.; Posthuma, D. MAGMA: Generalized Gene-Set Analysis of GWAS Data. *PLOS Computational Biology* **2015**, *11*, e1004219, doi:10.1371/journal.pcbi.1004219.
5. Human genomics. The Genotype-Tissue Expression (GTEx) pilot analysis: multitissue gene regulation in humans. *Science* **2015**, *348*, 648-660.
6. Kang, H.J.; Kawasawa, Y.I.; Cheng, F.; Zhu, Y.; Xu, X.; Li, M.; Sousa, A.M.; Pletikos, M.; Meyer, K.A.; Sedmak, G.; et al. Spatio-temporal transcriptome of the human brain. *Nature* **2011**, *478*, 483-489, doi:10.1038/nature10523.
7. Nagel, M.; Watanabe, K.; Stringer, S.; Posthuma, D.; van der Sluis, S. Item-level analyses reveal genetic heterogeneity in neuroticism. *Nat Commun* **2018**, *9*, 905, doi:10.1038/s41467-018-03242-8.
